# Comments on the article “high complication rate following dynamic intraligamentary stabilization for primary repair of the anterior cruciate ligament”: the story of the cyclops syndrome is not over

**DOI:** 10.1007/s00167-019-05527-x

**Published:** 2019-06-17

**Authors:** Christophe Trojani

**Affiliations:** Chirurgie Orthopédique, Unité de Chirurgie du Genou, Institut Universitaire Locomoteur Et du Sport (iULS), Hôpital Pasteur 2, CHU de Nice, 30 Avenue voie Romaine, 06100 Nice, France

Dear Editor-in-Chief,

I was very interested in reading the article "high complication rate following dynamic intraligamentary stabilization for primary repair of the anterior cruciate ligament” [11]. This article highlights the high rate of complications following primary repair of the anterior cruciate ligament (ACL) by dynamic intraligamentary stabilization (DIS). DIS may lead to healing of an acute ACL rupture [1], especially in case of ACL tear with an intact synovial coverage [2].

Among the described complications, the cyclops syndrome rate is top rank. This syndrome corresponds to a local arthrofibrosis and was described in 1990 as a loss of knee extension after ACL reconstruction due to the progressive development of a fibrovascular nodule at the tibial side of the ACL graft [6]. The term cyclops was chosen because of the eye-like appearance of this lesion on MRI and at arthroscopy, by analogy to the giants of Greek mythology with a single eye in the middle of their forehead. This syndrome includes a progressive loss of knee extension of 10°–20° in the first 6 months after ACL reconstruction, associated with anterior knee pain and a clunk when trying to extend the knee [4].

At the beginning of the twenty-first century, other authors have reported patients presenting a progressive loss of extension after partial or complete ACL tears not treated by surgical reconstruction [9, 14–16]. If associated with a partial ACL tear, the knee is not unstable with a negative pivot shift because of the loss of extension, and patients show an average 10° loss of extension. During arthroscopy, a cyclops nodule is easily found when associated with a partial ACL tear, and isolated removal of the cyclops is mandatory. On the contrary, if associated with a complete ACL tear, the cyclops nodule may be hidden in the fat pad. Removal of the cyclops associated with ACL reconstruction is necessary to prevent extension deficit.

More recently, it has been shown that augmentation ACL procedures with preservation of the ACL remnant fibers [12, 13] and DIS procedures for primary repair of the ACL [7, 10] could lead to a high rate of cyclops syndrome. Augmentation procedures [5] and primary repair [17] are old techniques for ACL tears, and it is well known since more than 30 years, that an augmentation procedure may lead to a mechanical block of extension [5]. One could say that the recent revival of augmentation or primary repair for ACL tears [3, 8], also leads to the revival of well-known complications.

This letter to the editor is therefore intended to recall the potentially high rate of cyclops syndrome encountered with ACL primary repair or augmentation and to alert on the paradox of the cyclops syndrome’s recent return, due to the recent revival of old techniques for ACL tears. In conclusion, the story of the cyclops syndrome is not over: it may persist after ACL reconstruction, it is not well known after a partial or complete ACL tear and its revival due to ACL repair, or augmentation techniques is a concern.

## References

[1] Ahmad SS, Schreiner AJ, Hirschmann MT, Schröter S, Döbele S, Ahrend MD, Stöckle U, Ateschrang A (2019). Dynamic intraligamentary stabilization for ACL repair: a systematic review. Knee Surg Sports Traumatol Arthrosc.

[2] Ateschrang A, Schreiner AJ, Ahmad SS, Schröter S, Hirschmann MT, Körner D, Kohl S, Stöckle U, Ahrend MD (2019). Improved results of ACL primary repair in one-part tears with intact synovial coverage. Knee Surg Sports Traumatol Arthrosc.

[3] Colombet P (2018). Editorial commentary: anterior cruciate ligament augmentation: a bold, technically demanding surgical technique… but don't forget to evaluate the benefit-risk ratio. Arthroscopy.

[4] Delincé P, Krallis P, Descamp PY (1998). Different aspects of the Cyclops lesion following anterior cruciate ligament reconstruction: a multifactorial pathogenesis. Arthroscopy.

[5] Fullerton LR, Andrews JR (1984). Mechanical block to extension following augmentation of the anterior cruciate ligament. Am J Sports Med.

[6] Jackson DW, Schaefer RK (1990). Cyclops syndrome: loss of extension following intraarticular anterior cruciate ligament reconstruction. Arthroscopy.

[7] Kohl S, Evangelopoulos DS, Schär MO, Bieri K, Müller T, Ahmad SS (2016) Dynamic intraligamentary stabilisation: initial experience with treatment of acute ACL ruptures. Bone Joint J 98-B(6):793–798 10.1302/0301-620X.98B6.35040 27235522

[8] Mahapatra P, Horriat S, Anand BS (2018). Anterior cruciate ligament repair—past, present, and future. J Exp Orthop.

[9] Mc Mahon PJ, Bowen MK, Warren RF (1999). The cyclops lesion: a cause of diminished knee extension after rupture of the anterior cruciate ligament. Arthroscopy.

[10] Meister M, Koch J, Amsler F, Arnold MP, Hirschmann MT (2018). ACL suturing using dynamic intraligamentary stabilisation showing good clinical outcome but a high reoperation rate: a retrospective independent study. Knee Surg Sports Traumatol Arthrosc..

[11] Osti M, El Attal R, Doskar W, Höck P, Smekal V (2019). High complication rate following dynamic intraligamentary stabilization for primary repair of the anterior cruciate ligament. Knee Surg Sports Traumatol Arthrosc.

[12] Sonnery-Cottet B, Lavoie F, Ogassawara R, Scussiato RG, Kidder JF, Chambat P (2010). Selective anteromedial bundle reconstruction in partial ACL tears: a series of 36 patients with mean 24 months follow-up. Knee Surg Sports Traumatol Arthrosc.

[13] Sonnery-Cottet B, Panisset JC, Colombet P, Cucurulo T, Graveleau N, Hulet C, Potel JF, Servien E, Trojani C, Djian P, Pujol N, French Arthroscopy Society (SFA) (2012). Partial ACL reconstruction with preservation of the posterolateral bundle. Orthop Traumatol Surg Res.

[14] Tonin M, Saciri V, Veselko M, Rotter A (2001). Progressive loss of knee extension after injury: cyclops syndrome due to a lesion of the anterior cruciate ligament. Am J Sports Med.

[15] Trojani C, Coste JS, Michiels JF, Boileau P (2003). Le syndrome du cyclope : un problème déjà présent avant la reconstruction arthroscopique du ligament croisé antérieur. J Trauma Sport.

[16] Veselko M, Rotter A, Tonin M (2000). Cyclops syndrome occurring after partial rupture of the anterior cruciate ligament not treated by surgical reconstruction. Arthroscopy.

[17] Weaver JK, Derkash RS, Freeman JR, Kirk RE, Oden RR, Matyas J (1985). Primary knee ligament repair—revisited. Clin Orthop Relat Res.

